# Evaluation of gene importance in microarray data based upon probability of selection

**DOI:** 10.1186/1471-2105-6-67

**Published:** 2005-03-22

**Authors:** Li M Fu, Casey S Fu-Liu

**Affiliations:** 1 Pacific Tuberculosis and Cancer Research Organization, Pasadena, California, USA; 2 University of Florida, Gainesville, Florida, USA

## Abstract

**Background:**

Microarray devices permit a genome-scale evaluation of gene function. This technology has catalyzed biomedical research and development in recent years. As many important diseases can be traced down to the gene level, a long-standing research problem is to identify specific gene expression patterns linking to metabolic characteristics that contribute to disease development and progression. The microarray approach offers an expedited solution to this problem. However, it has posed a challenging issue to recognize disease-related genes expression patterns embedded in the microarray data. In selecting a small set of biologically significant genes for classifier design, the nature of high data dimensionality inherent in this problem creates substantial amount of uncertainty.

**Results:**

Here we present a model for probability analysis of selected genes in order to determine their importance. Our contribution is that we show how to derive the *P *value of each selected gene in multiple gene selection trials based on different combinations of data samples and how to conduct a reliability analysis accordingly. The importance of a gene is indicated by its associated *P *value in that a smaller value implies higher information content from information theory. On the microarray data concerning the subtype classification of small round blue cell tumors, we demonstrate that the method is capable of finding the smallest set of genes (19 genes) with optimal classification performance, compared with results reported in the literature.

**Conclusion:**

In classifier design based on microarray data, the probability value derived from gene selection based on multiple combinations of data samples enables an effective mechanism for reducing the tendency of fitting local data particularities.

## Background

Based on the concept of simultaneously studying the expression of a large number of genes, a DNA microarray is a chip on which numerous probes are placed for hybridization with a tissue sample. The DNA microarray has recently emerged as a powerful tool in molecular biology research, offering high throughput analysis of gene expression on a genomic scale. However, biological complexity encoded by a deluge of microarray data is being translated into all sorts of computational, statistical or mathematical problems.

Driven by the growing genomic technology, molecular medicine has become a rapidly advancing field. An important research topic is to identify disease-related gene expression patterns based on microarray analysis. In one approach, genes are selected for constructing a clinically useful classifier for disease diagnosis. The genes thus selected often shed light on the fundamental molecular mechanisms of the disease [1]. As addressed in several research works [1-5], the problem of gene selection considered in this context is a difficult one because there are thousands of genes at hand but only a very limited number of samples are available. Mathematically, this problem is characterized by high data dimensionality. To develop a classifier, dimensionality reduction by gene selection is essential. Genes selected for constructing a classifier are believed to be important. Typically, only a small fraction of genes differentially expressed in the diseased tissue will be selected.

There exist two related but different objectives for gene selection. As mentioned above, one objective is to construct a classifier or predictor for classifying, diagnosing, or predicting the type of cancer tissue according to the expression pattern of selected genes in the tissue [6]. The other objective is to determine whether the changes in gene expression across two conditions are significant (e.g., SAM) [7]. The present work is developed in the first context.

Here, we report new theoretical developments and research results as an extension of our earlier work [4,8], presenting a new probabilistic analysis of gene selection from microarray data, which distinguishes our work from other related work.

## Results

### Probability analysis of selected genes

Under very high data dimensionality, questions can be raised of whether genes could have been selected by chance and whether selected genes are sufficiently significant beyond any doubt due to inherent uncertainty or data particularity. Quite often, not identical sets of genes are selected from different subsets of the data. At the fundamental level, it would be important to distinguish between the case of diverse patterns and the case of false patterns. To address the problem, we take the approach that takes into account both statistical significance and performance issues. The bootstrapping technique lends itself well as far as the first issue is concerned.

Suppose we randomly draw samples from a given domain and conduct a gene selection experiment. Assume that we select one gene out of a total of *p *genes. The probability of the event that a particular gene is selected in a single trial is *1/p*. According to the information theory, the smaller the probability is, the more informative the event is. Given a large *p*, it seems that the event is significant, and this would be true only if we have a particular gene in mind before gene selection; otherwise, the probability should be adjusted for the presence of *p *genes, and then it becomes clear that any gene selected in a single trial is non-informative. Now suppose we conduct multiple trials and ask the question of whether any gene repeatedly selected across trials is significant. Here we devise an analysis for the question.

#### Theorem

In *r *multiple independent trials conducted for gene selection, select one gene out of a total of *p *genes in each trial. Given the level of significance α, a gene is considered significant if it is selected *r *times in *r *trials and



#### Proof

The probability of the event that the same gene is selected *r *times in *r *trials is (1/*p*)^*r*^. Since there are *p *genes, the adjusted probability (analogous to Bonferroni's correction) is *p*(1/*p*)^*r*^. Therefore,



Equivalently,



Thus,



Note that the value of  is negative. The result follows. €

#### Corollary 1

The minimum threshold value of *r *for reaching the given level of significance is



where ⌈⌉ is the ceiling operator. This is because *r *must be an integer greater than or equal to the real threshold.

For example, consider the leukemia data [1]. There are 7129 genes. Assume α = 0.05. From Eq. (1), *r*_*θ *_= 2.

Consider a more general case: what is the probability of the event that a gene is selected *r *times in *m *trials? The adjusted probability becomes



where  is the combinatorial function that returns the number of possibilities for choosing *r *from *m *objects. Assume a large *p *so that. Then, we have



The level of significance (α in Eq. (1) and (2)) is set to 0.05 by convention in the present work.

### Reliability analysis of gene selection

The innovative feature of our method is to conduct reliability analysis for arriving at the gene expression signature. The analysis assesses the repeatability of genes selected and determines the repeatability for gene selection using *M*-fold cross-validation.

In the 10-fold cross-validation approach, the data set is divided into 10 disjoint subsets of about equal size. Genes are selected on the basis of nine of these subsets, and then the remaining subset is used to estimate the predictive error of the trained classifier using only the selected genes. This process is repeated 10 times, each time leaving one set out for testing and the others for training. The cross-validation error rate is given by the average of the 10 estimates of the error rate thus obtained.

In each cross-validation cycle, we conduct SVM-RFE gene ranking and selection operations, as described in the Methods section. We select a minimal set of genes by collecting from the top rank one by one and picking the set associated with minimum error in each cross-validation cycle. There is no guarantee that the same subset of genes will be selected in each of the 10 cycles in 10-fold cross-validation. However, vital genes tend to be selected more consistently than others across cycles. The significance of a gene is correlated with the repeatability of selection according to the probabilistic analysis given earlier. We associate each selected gene with a repeatability value indicating how many times it is selected in the cross-validation experiment. The biological or clinical interpretation of "repeatability" would depend on the objective and design of the microarray experiment. We may consider the validity of a selected gene by its reliability in the sense that the more often a gene is selected, the less likely chance is a factor.

To select the final set of genes, we need to determine the repeatability threshold. A gene is in the final set if its repeatability reaches (i.e., no less than) the threshold. To this end, second 10-fold cross-validation is performed. Then we choose the repeatability threshold that is associated with the minimal cross-validation error under the given level of significance (α = 0.05). Recall that a gene with a higher repeatability is associated with a small *P *value, as shown earlier.

To extend the method from two-class to multi-class classification, we adopt the one-against-all others strategy under which genes are selected for each class one at a time and then combined. For each class, all the other classes are grouped as a single class. In this way, a multi-class gene selection problem is converted into a series of two-class problems. The program was written in Matlab [9]. An SVM Matlab toolbox as well as Mathlab is required for the program use.

### Case analyses

In cancer research, our current goal is to develop a molecular classifier based on tissue gene expression patterns for diagnosis and subtype classification. With this in mind, we evaluate our method using well-known benchmark microarray data sets including those concerning small round blue cell tumors, colon cancer, leukemia as well as perturbed data sets.

The small round blue cell tumors (SRBCTs) data set includes 63 training samples and 25 test samples derived from both tumor biopsy and cell lines [10]. In consistency with other reports in the literature, we used the test set of 20 samples after 5 non-SRBCT samples were removed. The data set consists of four types of tumor in childhood, including Ewing's sarcoma (EWS), rhabdomyosarcoma (RMS), neuroblastoma (NB), and Burkitt lymphoma (BL). After initial screening, the data set in the public domain contains 2308 genes.

The colon cancer data set contains 62 tissue samples, each with 2000 gene expression values [11]. The tissue samples include 22 normal and 40 colon cancer cases. In this study, we used all the 62 samples in the original data.

The leukemia data consist of 72 tissue samples, each with 7129 genes expression values [1]. The samples include 47 ALL (acute lymphoblastic leukemia) and 25 AML (acute myeloid leukemia). The original data have been divided into a training set of 38 samples and a test set of 34 samples.

The reference method with which we compared our method applied a technique referred to as SVM-RFE [3] to select genes from the training data without reliability assessment. The reference method [12] is a multi-class extension of the SVM-RFE method used for two-class classification. The SVM-RFE method (two-class or multi-class) has not been applied to the SRBCT data before. We implemented the computer algorithm of the reference method for comparison with ours. The same experimental conditions were applied to both methods.

### Small round blue cell tumor classification

On the SRBCT data, our method selected 19 genes (Table 1) from the microarray gene expression data of the 63 training samples. The SVM classifier trained on the 63 training samples using the 19 selected genes was tested on the 20 different test samples. Both the training and test predictive accuracies were 100%. That is, the trained SVM classifier can accurately predict the tumor class using the 19 gene expression data for both seen and unseen samples. Since the classifier may tend to fit the training data, the generalization performance of the classifier is indicated by the test accuracy.

The reference method selected 8 genes with 100% training accuracy but with only 90% test accuracy. It seemed that the reference method did not select enough genes even though the selected genes could correctly classify all the training samples – an example of over-generalization, whereas our bootstrap-like strategy adequately dealt with this problem by taking into account of both reliability and diversity in gene selection.

We examined the consensus of genes selected by our method and by two other best-known methods: the method of Khan et al. [10] based on artificial neural networks and the method of Tibshirani et al. [13] based on shrunken centroids, and we found that there was high consensus between our and their results. Out of the 19 genes selected by our method, 18 genes were also selected by Khan's method and 16 genes by Tibshirani's method (Table 1). While agreement among results produced by different methods may imply similarities in the inductive biases, these two other methods use fundamentally different representational biases. Thus, such agreement should not be taken for granted and would instead serve as substantial evidence indicative of the validity and significance of our method.

**Figure 1:**
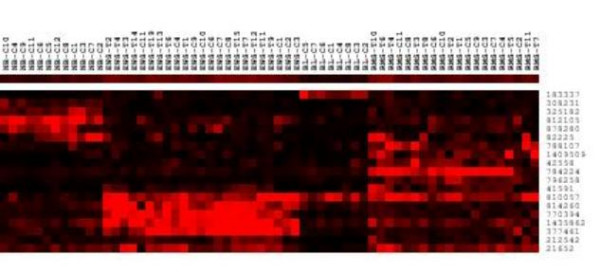
The gene expression map of the 19 genes selected by our method in the domain concerning classification of SRBCTs. The map was generated by Eisen's hierarchical clustering program called CLUSTER and viewed by the TREEVIEW program. Four sample clusters are visually recognizable, corresponding exactly to the four predefined tumor classes (NB, EWS, BL, and RMS) with 100% accuracy.

Whether the selected genes served as meaningful markers for cancer classification was further confirmed by cluster analysis and visualization. To this end, we applied a hierarchical clustering program developed by Eisen [14] to the gene expression data of the selected genes. By visual inspection of the gene expression map, four clearly separated clusters (Figure 1) were identified. Upon verification, each cluster corresponded exactly to a distinct tumor group with 100% accuracy. Thus, a diagnostic chip can be designed based on the selected genes. This result also provides additional evidence to support our method.

### Colon cancer diagnosis

In performance analysis, we conducted multiple experiments with random data partitions. In each experiment, the data were randomly and equally split into training and test sets. The training set was used for gene selection and classifier training, and the test set for determining the predictive performance of the classifier based on the genes selected by the given algorithm. Our method outperformed the reference method by a small margin. This result reflects the underlying fact that there are multiple possible ways of selecting genes for constructing a classifier with comparable performance using different methods.

**Figure 2:**
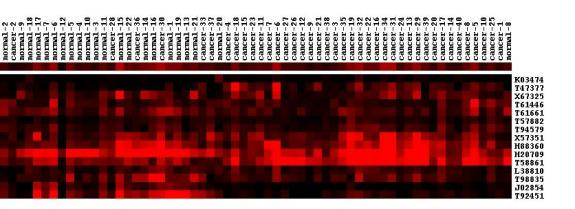
The gene expression map of the 15 genes selected from the colon cancer microarray data set using our method. Two major sample clusters can be recognized by visual inspection, corresponding to normal and cancer tissue samples, respectively.

Our program selected 15 genes from the colon cancer data (Table 2). The selected genes allow the separation of cancer from normal samples in the gene expression map (Figure 2, Table 3). Some genes were selected because their activities resulted in the difference in the tissue composition between normal and cancer tissue. Other genes were selected because they played a role in cancer formation or cell proliferation. It was not surprise that some genes implicated in other types of cancer such as breast and prostate cancers were identified in the context of colon cancer because these tissue types shared similarity.

Our method is supported by the meaningful biological interpretation of selected genes, as discussed below. New biological hypotheses can be formulated to further investigate the relationship of a particular gene with colon cancer. For example, what is the role of profilin 1 protein in colon cancer? Some discovered genes could potentially serve as novel targets for drugs, vaccines, or gene therapy.

### Leukemia classification

On the leukemia data, our method selected four genes (Table 4) from the microarray gene expression data of 38 training samples. The SVM classifier trained on the 38 training samples using the selected genes was tested on the 34 different test samples. The training and test accuracies were 100% and 97.06%, respectively. In addition, the AML and ALL samples formed separate clusters in the gene expression map of the selected genes.

The reference method also selected four genes and achieved the same level of test accuracy as our method. The original algorithm of SVM-RFE [3] selected 8 or 16 genes on this data set. The method based on shrunken centroids [13] selected 21 genes on this data set. A recent study indicated that the unbiased error estimate of the classifier using a small number of selected genes was virtually non-zero on the leukemia data set [6]. Taken together, the evidence showed that our method produced optimum results in terms of both predictive performance and the number of selected genes.

### Perturbed data

In practical circumstances, noise may arise during sample collection and handling, slide preparation, hybridization, or image analysis, as reflected by variations in microarray results generated from different laboratories. To address this issue, we also conducted performance evaluation of our gene selection method based on perturbed data. 20 data sets were produced by randomly perturbing 5% (rounded up to the nearest integer) of the training cases, reversing their class labels and leaving the test cases intact, in the domains of colon cancer diagnosis and leukemia classification (ten in each domain). The average test predictive accuracies with our method in the two domains were 85.49% and 88.61%, respectively, compared with 80.65% and 86.11% with the reference method. The result suggests the potential advantage with our method in smoothing out data variations due to various sources in practice.

## Discussion

Both cross-validation and bootstrapping are standard statistical methods for arriving at an unbiased estimate of the true error rate associated with a classifying or predicting system. Bootstrapping has also been used for assessing the reliability or stability of phylogenetic trees [15] or cluster analysis [16]. Bootstrapping is a method for random re-sampling with replacement for a number of times and estimates the error rate by the average error rate over the number of iterations. Cross-validation is a method of assessing the reliability of error; however, its application to learning the pattern in the data is novel. As discussed later, stability emerges as an important issue in gene selection. Here we propose to use bootstrapping or cross-validation for analyzing the issue. Our experience showed that cross-validation was more efficient than bootstrapping. For instance, genes selected based on a single 10-fold cross-validation were more accurate in prediction than those selected using bootstrapping with 10 re-sampling iterations. Since the SVM-based gene selection algorithm is time-consuming, we consider only cross-validation for assessment of error and stability in this study.

In the original SVM-RFE algorithm [3], error estimation and gene selection are not independent processes because both are based on the same training set. However, it is important to correct for the selection bias by performing a cross-validation or applying a bootstrap external to the selection process [6,17]. Our implementation of SVM-RFE is based on this idea.

Genes selected for cancer diagnosis or classification can be validated by their biological significance since these genes are expected to show differential expression between normal and cancer tissue or among subtypes of cancer, and as such, they are implicated in cancer-related mechanisms or pathways. Genes with unknown roles may be discovered through gene selection and later verified by biological studies.

From the SRBCT data set, genes selected by our method for a particular type of cancer/tumor against other types are generally consistent with its tissue of origin. For example, genes selected for neuroblastoma (NB) are characteristic for nerve cells, such as neuronal N-cadherin, and meningioma 1; genes selected for rhabdomyosarcoma (RMS) are characteristic for muscle cells, such as alpha sarcoglycan, and slow skeletal troponin T1; genes selected for Burkitt lymphoma (BL) are characteristic for lymphocytes or blood cells, such as major histocompatibility complex (class II, DM alpha). Some genes discovered by means of microarray analysis have been reported in the biological literature, e.g., over-expression of MIC2 in Ewing's sarcoma (EWS) [18]. Some genes are over-expressed in a certain type of tumor but lack specificity. For instance, FGFR4 (fibroblast growth factor receptor 4) was noted to be highly expressed only in RMS and not in normal muscle, but it is also expressed in some other cancers and normal tissues [10]. A gene that is under-expressed in a particular type of tumor compared with other types can also be selected as a diagnostic marker. For instance, cold shock domain protein A selected for NB was under-expressed in this tumor, consistent with the fact that this gene is expressed in B cells and skeletal muscle but not in the brain [13].

With our method, four muscle-related genes (H20709, T57882, T92451, and J02854) were selected from the colon cancer data, reflecting the fact that normal colon tissue had higher muscle content, whereas colon cancer tissue had lower muscle content (biased toward epithelial cells) [11]. The selection of 60s ribosomal protein L30E agreed with an observation that ribosomal protein genes had lower expression in normal than in cancer colon tissue [11]. The selected interferon inducible protein 1-8D genes were found to be expressed in adenocarcinoma cell lines [19]. There was a potential connection of another selected gene, human chitotriosidase, to cancer [3]. The implications of cancer among other selected genes are explained as follows. S-100 protein can stimulate cellular proliferation and may function as a tumor growth factor [20]. Profilin 1 protein can suppress tumorigenicity in breast cancer cells. A study showed consistently lower profilin 1 levels in tumor cells [21]. The reduced expression of P27 protein was linked to the possibility of colon carcinoma [22]. The A20 protein can inhibit a specific apoptotic pathway [23]. Recall that apoptosis is a major mechanism for tumor suppression. The guanine nucleotide-binding protein is involved in signal transduction and its abnormality may contribute to cancer development [24]. A thyroid receptor interactor could be a target gene of a certain oncogene. The alpha trans-inducing protein (bovine herpesvirus type 1) may be linked to oncogenic activity.

In the related work [3], 7 genes were selected from the colon cancer data: H08393, M59040, T94579, H81558, R88740, T62947, and H64807. For all of them, a possible link to cancer was found in the biological literature. These 7 genes, however, do not include any muscle-specific gene, despite that muscle content offered a discriminating index for colon cancer [11].

In a typical microarray data analysis problem, the data dimensionality is high and the sample size is relatively small. Under this condition, the problem of finding a classification model is under-constrained, and the model found tends to fit the training data so closely that it fails to generalize to unseen data. To address the issue of data overfitting, the SVM has the capability of controlling the model complexity to the point where a satisfactory solution can be produced. On the other hand, the ability of causal discovery based on the SVM-RFE approach or an alternative approach is discounted by the finding that most genes selected are selected only once from one data split to another in *M*-fold cross-validation [25]. This means that the SVM is not free of the data-overfitting problem at least in the context of gene selection from microarray data, and it raises the question about stability or reliability of gene selection, as we address here.

The research finding that the SVM may assign zero weights to strongly relevant variables and non-weights to weakly relevant (red-herring) features [26] implies the disadvantage with this approach for discovery of causal variables associated with the target variable concerned. This however can be understood since the SVM-RFE is aimed to identify the best features for maximum margin of separation between different classes of samples, regardless of causal implications. In reality, causal variables are not necessarily most discriminant, as the target variable is not always categorized according to its causal factors. The issue of causality becomes even more complicated because of confounding variables leading to so-called spurious causation. The method presented here is developed in the context of cancer subtype classification and evaluated in terms of predictive performance rather than the capability of causal inference. However, some methods are both predictive and causal [26,27].

We emphasize the importance of holding back some data to improve generalization and diversity of the learning outcome. In application of *M*-fold cross-validation to *n *samples, *M *can assume a value ranging from 2 to *n*. A small *M *is not sufficient to assess the repeatability of selected genes while a large *M *(e.g., *M = n *in the leave-one-out experiment) is associated with high degree of redundancy on data for training and low diversity of genes selected. This argument suggests that there exists an optimum *M *value. So we conducted experiments to compare predictive accuracies for three cases: *M *= 5, 10, and 15. Among the three cases, 10-fold cross-validation achieved the best results. It is thus consistent with our intuitive analysis. However, there is no proof that 10-fold cross-validation is always the best choice. In practice, the optimum *M *value should be determined by the value associated with the best cross-validation accuracy.

This study highlights the importance of reliability assessment of genes selected from a large-scale microarray data. We show how to derive the *P *value of each selected gene in multiple gene selection trials based on different data partitions. The importance of a gene is indicated by its associated *P *value. The distinctive feature of our method is that gene selection is determined by both ranking and reliability analyses. Reliability analysis is conducted using *M*-fold cross-validation. Some gene selection methods [3,28] use cross-validation to determine the number of selected genes by minimum cross-validation error but not by optimum repeatability as in our method. Thus, reliability analysis comprising repeatability measurement and optimum repeatability determination defines the novelty of our method, which has enabled a more accurate and cost-effective cancer classifier to be constructed, compared with other methods. Notice, however, the argument about reliability or stability must rest on the assumption of sound performance, as will be clear from the apparent stability with some trivial approaches to gene selection such as the one based on lexicographic ordering of gene names. In fact, the theory behind the analytical scheme we developed is a general one and can therefore be extended to other performance-based gene selection methods.

## Conclusion

The DNA microarray technology has become a standard tool for gathering genome-wide gene expression information. Molecular classification based on gene expression information has emerged as an important approach to cancer diagnosis. A cost-effective approach is to select a small set of genes for classifier design. Moreover, it may be ineffective to use whole microarray data for classification purposes because the data dimensionality (i.e., the number of variables/genes) is often several orders of magnitude greater than the available sample size.

Experience shows that different sets of genes can be selected from different combinations of microarray data instances with the same gene selection algorithm. At the same time, it is noticed that a biologically significant gene tends to be selected repeatedly across different combinations of data instances. We have developed a method for analyzing this situation. In the domain of small round blue cell tumor subtype classification, we have demonstrated that the method we developed selected only 19 genes that provided 100% accuracy on both training and test data sets. In comparison, the approach based on artificial neural networks [10] selected 96 genes, and the shrunken centroid method [13] selected 43 genes. Thus, our method suggests a mechanism for effectively reducing the tendency of fitting local data particularities in the process of gene selection for classifier design based on microarray data.

## Methods

This section provides the details of the methods, but the novelty aspects are described in the "Results" section.

### Classification based on support vector machines

We use the method of support vector machines (SVM) [29,30] for classification. The SVM has been demonstrated as a useful tool for analyzing microarray data [31]. Consider *n *training samples {(, *y_i_*) | 1 ≤ *i *≤ *n *}, where , is the input feature vector for the *i*th sample and *y*_*i *_is the corresponding target class (output). The basic problem for training an SVM can be reformulated as: given a set of *n *training instances, each represented as (, *y_i_*), maximize



subject to



The optimal hyperplane that separates different classes of objects can be constructed from the solutions α_*I*_'s to this maximization problem. The SVM can perform a nonlinear transformation via the inner-product kernel  to map the input space into a new high-order feature space where the patterns are linearly separable with high probability. The use of such a kernel function can lead to a decision function that is non-linear in the input space but its image is linear in the transformed space. When the samples are not linearly separable, whether in the input or transformed space, a soft-margin algorithm as an extension of the basic algorithm is available [32].

The SVM used in this study employed the linear kernel since we found that it yielded a better result than a non-linear kernel for the data under investigation, and this observation is also consistent with the literature [3]. All SVM parameters were set to the standard values in accordance with the convention: s = 0 (C-SVM), t = 0, c = 100, v = 10.

Data normalization in the case of cDNA arrays proceeded as follows: the local background intensity is subtracted from the value of each spot on the array; the two channels are normalized against the median values on that array; the Cy5/Cy3 fluorescence ratios and log_10_-transformed ratios are calculated from the normalized values. In addition, genes that do not change significantly can be removed through a filter in a process called data filtration.

### Gene selection

An SVM-based gene selection algorithm has two main components: gene ranking and gene selection. Gene ranking results in a sorted list of genes in decreasing order of importance for classification. This issue is complicated since some genes become important only if combined with other genes. After genes are ranked, genes are selected according to their ranks.

When there are a large number of features, a conservative strategy is to determine the least important feature one at a time recursively. In this work, we adopted the SVM-RFE (recursive feature elimination) algorithm [3] where the least important feature is identified and removed in each iteration, remaining features are re-evaluated, and the process repeats until no more features are left for consideration. For the linear kernel, the importance of a feature is determined by the associated weight magnitude, and the least important feature refers to the one with the smallest weight value. SVM-RFE essentially implements the strategy of backward feature elimination. In principle, feature ranking becomes more accurate as less important features are removed successively. To improve the speed, a chunk of least important features was eliminated per step until there were 256 genes remained, from which point, one gene was remove per step. The RFE ranking criterion is given by

*Rank(g_i_) *<*Rank(g_j_) *⇔ *Order-of-Elimination(g_i_) *>*Order-of-Elimination(g_j_)*

That is, the later a gene is eliminated, the higher (smaller) rank it has. So, the first-rank gene is last removed.

## Authors' contributions

L. Fu developed the method and conducted the experiments. C. Fu-Liu interpreted the data. Both authors drafted, read, and approved the manuscript.

## Figures and Tables

**Table 1:** Genes selected by our method on the microarray dataset of small round blue-cells tumors. Those genes also selected using the methods of Tibshirani et al. [13] and Khan et al. [10] are respectively marked by the symbol •.

Image ID	*P *Value	Gene Description	Tibshirani et al.	Khan et al.
21652	2.3 × 10^-5^	catenin (cadherin-associated protein), alpha 1	•	•
878280	2.3 × 10^-5^	collapsin response mediator protein 1		•
377461	< 0.000001	caveolin 1, caveolae protein	•	•
325182	2.3 × 10^-5^	cadherin 2, N-cadherin (neuronal)	•	•
1435862	0.02	MIC2 surface antigen (CD99)	•	•
42558	0.02	L-arginine:glycine amidinotransferase	•	•
812105	< 0.000001	transmembrane protein	•	•
41591	< 0.000001	meningioma 1	•	•
810057	< 0.000001	cold shock domain protein A	•	
183337	0.02	major histocompatibility complex, class II, DM alpha	•	•
796258	< 0.000001	sarcoglycan, alpha	•	•
1409509	0.02	troponin T1, skeletal, slow	•	•
788107	< 0.000001	amphiphysin-like		•
770394	< 0.000001	Fc fragment of IgG, receptor, transporter, alpha	•	•
82225	0.02	secreted frizzled-related protein 1		•
814260	< 0.000001	follicular lymphoma variant translocation 1	•	•
784224	< 0.000001	fibroblast growth factor receptor 4	•	•
308163	2.3 × 10^-5^	ESTs	•	•
212542	< 0.000001	cDNA DKFZp586J2118	•	•

**Table 2:** 15 genes selected from the colon cancer microarray data set (62 samples) using our method.

Gene Accession #	*P *value	Definition
H20709	< 0.000001	myosin light chain alkali, smooth-muscle isoform
X57351	< 0.000001	interferon-inducible protein 1-8D
T94579	< 0.000001	human chitotriosidase precursor
T47377	< 0.000001	S-100P protein (human)
T98835	< 0.000001	alpha trans-inducing protein (bovine herpesvirus type 1)
T61661	3.0 × 10^-5^	profilin I (human)
X67325	3.0 × 10^-5^	H. sapiens p27
T58861	0.02	60s ribosomal protein L30E
T61446	0.02	putative DNA binding protein A20
H88360	0.02	guanine nucleotide-binding protein G(OLF), alpha subunit
L38810	0.02	Homo sapiens thyroid receptor interactor (TRIP1)
T57882	0.02	myosin heavy chain, nonmuscle type A
T92451	0.02	tropomyosin, fibroblast and epithelial muscle-type
J02854	0.02	myosin regulatory light chain 2, smooth muscle isoform
K03474	0.02	human mullerian inhibiting substance gene

**Table 3:** Diagnosis results of the colon cancer data samples based on 15 selected genes, in correspondence with the gene expression map.

Normal Tissue		Cancer Tissue	
**Sample**	**Diagnosis**	**Sample**	**Diagnosis**
Normal-01	normal	Cancer-01	cancer
Normal-02	normal	Cancer-02	normal
Normal-03	normal	Cancer-03	cancer
Normal-04	normal	Cancer-04	cancer
Normal-05	normal	Cancer-05	cancer
Normal-06	normal	Cancer-06	cancer
Normal-07	normal	Cancer-07	cancer
Normal-08	cancer	Cancer-08	cancer
Normal-09	normal	Cancer-09	cancer
Normal-10	normal	Cancer-10	cancer
Normal-11	normal	Cancer-11	cancer
Normal-12	normal	Cancer-12	cancer
Normal-13	normal	Cancer-13	cancer
Normal-14	normal	Cancer-14	cancer
Normal-15	normal	Cancer-15	cancer
Normal-16	normal	Cancer-16	cancer
Normal-17	normal	Cancer-17	cancer
Normal-18	normal	Cancer-18	cancer
Normal-19	normal	Cancer-19	cancer
Normal-20	cancer	Cancer-20	cancer
Normal-21	normal	Cancer-21	cancer
Normal-22	normal	Cancer-22	cancer
		Cancer-23	cancer
		Cancer-24	cancer
		Cancer-25	cancer
		Cancer-26	cancer
		Cancer-27	cancer
		Cancer-28	normal
		Cancer-29	cancer
		Cancer-30	normal
		Cancer-31	cancer
		Cancer-32	cancer
		Cancer-33	cancer
		Cancer-34	cancer
		Cancer-35	cancer
		Cancer-36	normal
		Cancer-37	cancer
		Cancer-38	cancer
		Cancer-39	cancer
		Cancer-40	cancer

**Table 4:** Genes selected by our method on the leukemia microarray dataset. Those genes also selected using the methods of Golub et al.[1] and SVM-RFE (the reference algorithm) are respectively marked by the symbol •.

Access Number	*P *Value	Gene Description	Golub et al.	SVM-RFE
M27891	< 0.000001	CST3 Cystatin C	•	•
Y00787	< 0.000001	INTERLEUKIN-8 PRECURSOR	•	•
M19507	0.006	MPO Myeloperoxidase		•
L20688	0.006	Ly-GDI		
